# Supplementary material for: Identification and functional analysis of non-coding regulatory small RNA FenSr3 in Bacillus amyloliquefaciens LPB-18
Source: PeerJ. 2023 May 15;11:e15236. doi: 10.7717/peerj.15236 (PMC10194069; doi:10.7717/peerj.15236)
Supplement: Supplemental Information 4 [file peerj-11-15236-s004.zip › KO/CK-vs-T1_map/map05222.html]

KEGG PATHWAY: Small cell lung cancer - Reference pathway


|  |  |
| --- | --- |
| **Small cell lung cancer - Reference pathway** |  |

[
Pathway menu
| Organism menu
| Pathway entry
| Show description
| User data mapping
]

Lung cancer is a leading cause of cancer death among men and women in industrialized countries. Small cell lung carcinoma (SCLC) is a highly aggressive neoplasm, which accounts for approximately 25% of all lung cancer cases. Molecular mechanisms altered in SCLC include induced expression of oncogene, MYC, and loss of tumorsuppressor genes, such as p53, PTEN, RB, and FHIT. The overexpression of MYC proteins in SCLC is largely a result of gene amplification. Such overexpression leads to more rapid proliferation and loss of terminal differentiation. Mutation or deletion of p53 or PTEN can lead to more rapid proliferation and reduced apoptosis. The retinoblastoma gene RB1 encodes a nuclear phosphoprotein that helps to regulate cell-cycle progression. The fragile histidine triad gene FHIT encodes the enzyme diadenosine triphosphate hydrolase, which is thought to have an indirect role in proapoptosis and cell-cycle control.

|  |  |  |
| --- | --- | --- |
| Reference pathway | 184% 150% 122% 100% 82% 67% 55% | 图片下载 |
